# Translation of upstream open reading frames in a model of neuronal differentiation

**DOI:** 10.1186/s12864-019-5775-1

**Published:** 2019-05-20

**Authors:** Caitlin M. Rodriguez, Sang Y. Chun, Ryan E. Mills, Peter K. Todd

**Affiliations:** 1 Department of Neurology, University of Michigan, Ann Arbor, MI USA; 2 Neuroscience Graduate Program, University of Michigan, Ann Arbor, MI USA; 3 Department of Computational Medicine and Bioinformatics, University of Michigan, Ann Arbor, MI 48109 USA; 4 Department of Human Genetics, University of Michigan, Ann Arbor, MI 48109 USA; 5 VA Ann Arbor Healthcare System, Ann Arbor, MI USA

**Keywords:** Translation, Ribosome profiling, Upstream open reading frame, Near-cognate start codon, 5′ untranslated region, Neuronal differentiation

## Abstract

**Background:**

Upstream open reading frames (uORFs) initiate translation within mRNA 5′ leaders, and have the potential to alter main coding sequence (CDS) translation on transcripts in which they reside. Ribosome profiling (RP) studies suggest that translating ribosomes are pervasive within 5′ leaders across model systems. However, the significance of this observation remains unclear. To explore a role for uORF usage in a model of neuronal differentiation, we performed RP on undifferentiated and differentiated human neuroblastoma cells.

**Results:**

Using a spectral coherence algorithm (SPECtre), we identify 4954 consistently translated uORFs across 31% of all neuroblastoma transcripts. These uORFs predominantly utilize non-AUG initiation codons and exhibit translational efficiencies (TE) comparable to annotated coding regions. On a population basis, the global impact of both AUG and non-AUG initiated uORFs on basal CDS translation were small, even when analysis is limited to conserved and consistently translated uORFs. However, uORFs did alter the translation of a subset of genes, including the Diamond-Blackfan Anemia associated ribosomal gene *RPS24*. With retinoic acid induced differentiation, we observed an overall positive correlation in translational shifts between uORF/CDS pairs. However, CDSs downstream of uORFs show smaller shifts in TE with differentiation relative to CDSs without a predicted uORF, suggesting that uORF translation buffers cell state dependent fluctuations in CDS translation.

**Conclusion:**

This work provides insights into the dynamic relationships and potential regulatory functions of uORF/CDS pairs in a model of neuronal differentiation.

**Electronic supplementary material:**

The online version of this article (10.1186/s12864-019-5775-1) contains supplementary material, which is available to authorized users.

## Background

Alterations in protein expression and abundance are required for successful and stable cellular differentiation [1]. While changes in mRNA levels provide a partial view of networks driving such cellular changes, differences in translational efficiency (TE) act as an independent contributor to this process [2]. Determining ribosomal occupancy across the transcriptome through ribosomal profiling (RP) provides us with a powerful tool for assessing the relationship between mRNA abundance and translational output [3]. In particular, RP in cells and organisms has revealed a detailed picture of condition-specific changes in mRNA translation rates in multiple cellular processes from meiosis to development [4, 5].

The 5′ leader (traditionally referred to as the 5′ untranslated region) of mRNAs are one well-studied source of protein synthesis regulation [6–9]. 5′ leaders can regulate the synthesis of the main coding sequence (CDS) product through a variety of mechanisms [6, 9]. RNA secondary structures can impede ribosomal scanning, which decreases access of assembled 40S ribosomal preinitiation complexes to CDS initiation sites. Translation can also initiate within 5′ leaders at upstream open reading frames (uORFS). In the case of uORFs that terminate after the CDS initiation site (overlapping uORFs or “oORFs”), initiation in the 5′ leader directly competes with CDS initiation for scanning 40S ribosomes and is thus predicted to be inhibitory on CDS translation. In contrast, with uORFs that terminate within the 5′ leader and before the CDS initiation site (contained uORFs or “cORFs”), ribosomes can potentially reinitiate at the CDS. Thus, cORFs sometimes bypass other 5′ leader regulatory elements and can even provide stimulatory effects on CDS translation, but may be repressive as well. uORF translation can also indirectly influence CDS translation by influencing mRNA stability [10] or through interactions of newly synthesized uORF protein products with the translating ribosome [11, 12]. As such, the relationship of each uORF to the translation of its cognate CDS can be complex, making it difficult to define their specific functions and regulation across the transcriptome based on position alone.

Early ribosome profiling reports demonstrated that ribosome protected fragments (RPFs) are highly enriched within 5′ leader regions of mRNAs [3–5]. Since these first reports, there have been several studies that investigated 5′ leader translation [13–18]. These studies revealed potential roles for uORFs in circadian clock regulation, organism development, and the cell cycle [4, 19–22]. For example, AUG initiated uORFs were detected in the transcripts of key developmental signaling proteins during murine development [22]. Homozygous deletion of an AUG initiated uORF in the 5′ leader of *PTCH1*—which encodes the major receptor for SHH signaling—disrupted differentiation of mouse embryonic stem cells into neural progenitors [22]. Interestingly, ribosome profiling at various time points throughout neuronal differentiation of human embryonic stem cells revealed shifts in 5′ leader coverage on a number of transcripts [23]. However, these data were not systematically analyzed for active translation and characterization of uORFs, and relied solely on RPF reads as a measure translation of the whole 5′ leader. Additionally, few studies to date have included non-AUG initiated uORFs in their analysis [4, 24, 25] despite their potential to contribute significantly to the pool of footprints within 5′ leaders.

Treatment of human neuroblastoma cells with retinoic acid triggers their exit from the cell cycle and their differentiation into a neuron-like cell type [26, 27]. While many studies have sought to understand genetic changes underlying this process, most have focused on transcript-level changes, with evaluation of shifts in protein abundance only studied on a case-by-case basis [28–31]. Here we used RP in this simple model system to study the role of uORF activity in regulating protein translation during retinoic acid induced neuronal differentiation of neuroblastoma cells. Using a spectral coherence algorithm (SPECtre) and stringent dataset filtering we defined a set of translated uORFs, the majority of which initiate at a near-cognate start codon [14]. The presence of an AUG or conserved non-AUG uORF predicted lower CDS TE independent of cell state. Moreover, there were significant shifts in uORF usage that occurred with differentiation, suggesting a potential regulatory role. We observe less of a differentiation-dependent shift in translation of CDSs downstream of a uORF expressed across conditions, suggesting that uORFs act as a translational buffer on the transcripts in which they reside. Together this work provides important insights into how uORFs may function to regulate the translation of their associated CDS in a model of neuronal differentiation.

## Results

### Ribosome profiling detects conditionally regulated translation with differentiation

**Fig. 1:**
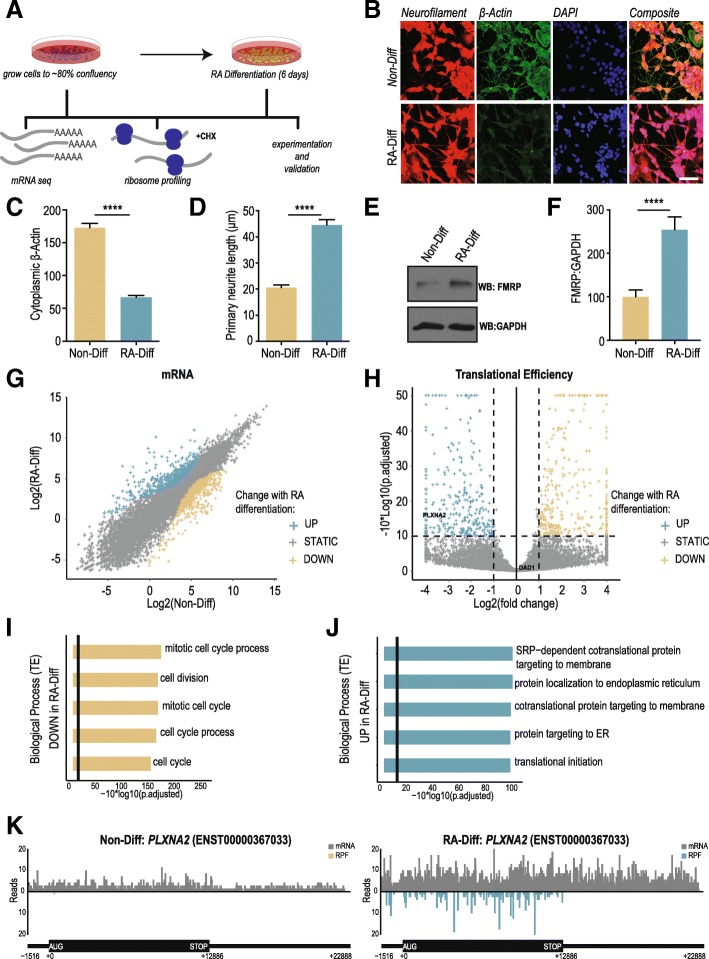
Retinoic acid treatment induces differential translation in SH-SY5Y human neuroblastoma cells. **a** Schematic of experimental design and data acquisition work-flow. **b** Immunocytochemistry performed on Non-Diff and RA-Diff SH-SY5Y cells with antibodies against neurofilament (red) and β-actin (green). Nuclei were DAPI stained (blue). **c** β-actin expression was decreased in RA-Diff cells. Individual cell fluorescence was quantified and represented as a corrected total cell fluorescence (CTCF) for β-actin; *n* = 119 for Non-Diff and *n* = 118 for RA-Diff. **d** Primary neurite length measured by neurofilament staining; *n* = 109 for Non-Diff and *n* = 100 for RA-Diff. **e** FMRP expression by immunoblot before and after RA treatment, quantified in F); *n* = 4 for both conditions. For panels C), D), and **f** Student’s t test, ****p ≤ 0.0001. Graphs represent mean ± S.E.M. **g** Differential mRNA abundance based on Non-Diff versus RA-Diff TPM. Transcripts were defined as significantly up-regulated (cyan) or down-regulated (gold) in the RA-Diff condition based on rank-change in abundance compared to the Non-Diff condition. **h** Volcano plot of transcripts with differential translation by translational efficiency (TE) by Riborex analysis. Significantly up-regulated genes (cyan) and down-regulated genes (gold) in RA-Diff cells are defined by an absolute log2-normalized fold-change cutoff of ±1 (vertical lines), and a multiple testing corrected *p*-value cutoff of 0.1 (horizontal line). **i** Gene sets (biological process) with significantly downregulated TE in RA-Diff cells. Genes upregulated in RA-Diff cells is shown in (**j**). The top five biological processes with significant change using a multiple testing corrected p-value cutoff of 0.05 (vertical line) are shown on the graph. **k** Plot shows normalized mRNA reads (grey) and RPF (cyan/gold) over the 5’leader (thin line, left), and CDS (thick line, middle). The axon guidance gene, *PLXNA2*, is representative of a transcript with higher translational efficiency and RPF in the RA-Diff condition.

We first confirmed the efficacy of RA treatment in differentiating SH-SY5Y cells. Cells were propagated to 80% confluency prior to 10 μM RA treatment for six days (Fig. 1a). RA treatment induces an exit from the cell cycle and a change in cellular morphology. Previous studies have used a similar protocol as a model for dopaminergic neuronal differentiation, although RA treatment is thought to generate a more immature neuron-like cell than what can be achieved from a neural progenitor [26, 27, 30]. Cytoskeletal alterations confirm a shift towards a more neuron-like state after 6 days of treatment. Cytoplasmic beta-actin immunofluorescence decreased and neurofilament labeled neurites increased in length in the differentiated cells (Fig. 1b-d) [32, 33]. We also observed an increase in expression of FMRP, a protein involved in neuronal function and translational control that is highly expressed in neurons relative to other cell types and tissues (Fig. 1e-f) [34]. Global mRNA sequencing (mRNA-Seq) demonstrated the anticipated transcriptional differences in RA Differentiated (“RA-Diff”) cells compared to in undifferentiated (“Non-Diff”) cells (Fig. 1g, Additional file 1: Table S1). Specifically, Gene ontology (GO) analysis revealed downregulation of transcript networks associated with mitotically active cells and upregulation of biological pathways associated with cell communication and stimulus response in the RA-Diff cells, consistent with exit from the cell cycle and transition to a more neuron-like state (Additional file 5: Figure S1).

Ribosome profiling can resolve the specific regions of mRNA undergoing translation at nucleotide resolution across the transcriptome within a cell population [3]. By comparing ribosomal occupancy within a given transcript in Non-Diff to RA-Diff cells, we are able to gauge translational differences. This can be accomplished by normalizing RPF abundance in the CDS to mRNA expression in samples prepared in parallel as a measure of TE (Fig. 1h). Inspection of biological processes by GO analysis with significant translational efficiency changes revealed mainly a downregulation of transcripts encoding proteins involved in mitosis in the RA Diff cells (Fig. 1i). Transcripts involved in endoplasmic reticulum (ER) function were significantly upregulated in the RA Diff cells (Fig. 1j). Investigation of transcript groups associated with a specific molecular function or cellular compartment further clarify the translational changes associated with differentiation (Additional file 5: Figure S2A-D). A more complete view of translation on an individual transcript is exemplified by *PLXNA2* which encodes a membrane-bound protein involved in nervous system development and axon guidance [35]. Its mRNA coverage is upregulated upon differentiation; however, the increased expression is much greater at the RPF level (Fig. 1k), producing a higher translational efficiency. Other transcripts such as *DAD1,* a factor critical for N-terminal glycosylation with roles in apoptosis and the unfolded protein response, exhibit shifts at both the mRNA and RPF level which produces no significant change in translational efficiency (Additional file 5: Figure S2E) [36]. All mRNA, RPF, and TE changes are detailed in Additional file 2: Table S2, and all GO analysis results are detailed in Additional file 3: Table S3.

### Characterization and experimental validation of SPECtre-identified uORFs

**Fig. 2:**
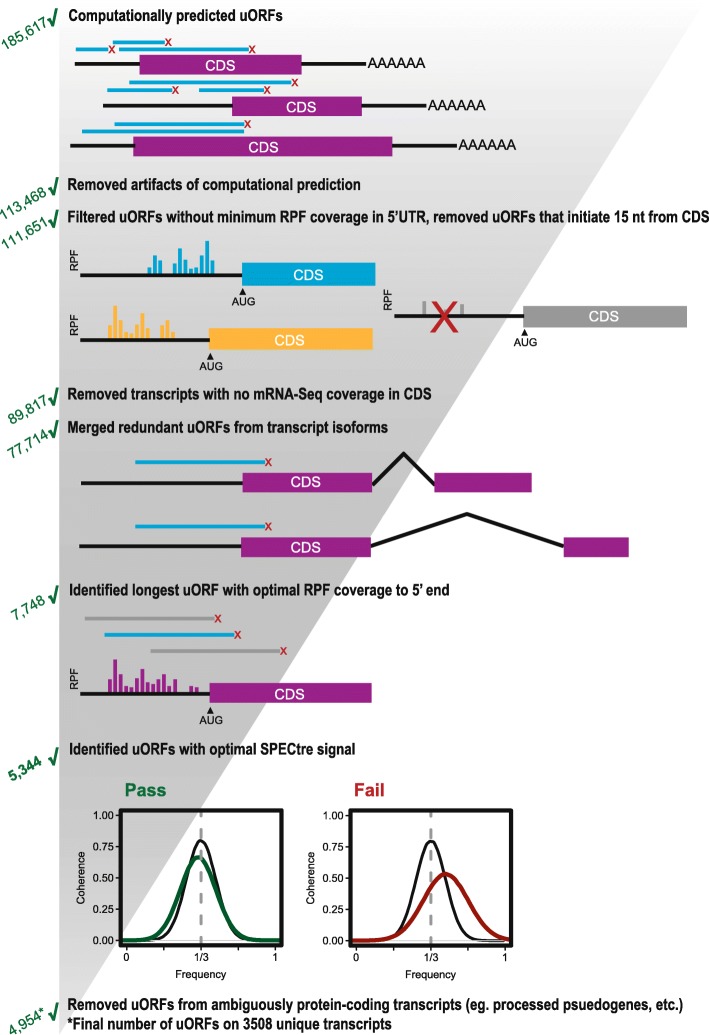
Computational prediction and filtering of upstream-initiated open-reading frames. ORFs were predicted from AUG and non-AUG, near-cognate translation initiation sites in the 5′ leader of annotated protein-coding genes, and computationally extended to the first termination site encountered in the 5′ leader (upstream-terminated ORFs) or CDS (overlapping ORFs). Predicted ORFs were then screened through a series of heuristic filters including: 1) minimum RPF coverage in the 5′ leader, 2) minimum mRNA-seq coverage in CDS, 3) in-frame N-terminal extensions, 4) redundant isoforms, 5) minimum length with optimal RPF coverage, 6) sufficient SPECtre signal, and 7) removal of ambiguously annotated protein-coding transcripts.

To annotate uORF sequences within the 5′ leader of mRNA, we utilized the SPECtre algorithm for classifying active regions of translation [14]. SPECtre accounts for the fundamental attribute of an actively translating ribosome to shift position three nucleotides at a time as it synthesizes new peptides and the ability of ribosome profiling to resolve this behavior with peaks in read coverage. Our algorithm takes an unbiased approach to scoring all potential uORFs from start site to the next in-frame stop codon (Fig. 2). For each potential uORF, the pattern of read coverage within this designated sequence is compared against the pattern of reads across all known protein-coding regions in the experimental library. This analysis results in a set of experimentally determined scores that are then subjected to a range of transcript-level filters.

**Fig. 3:**
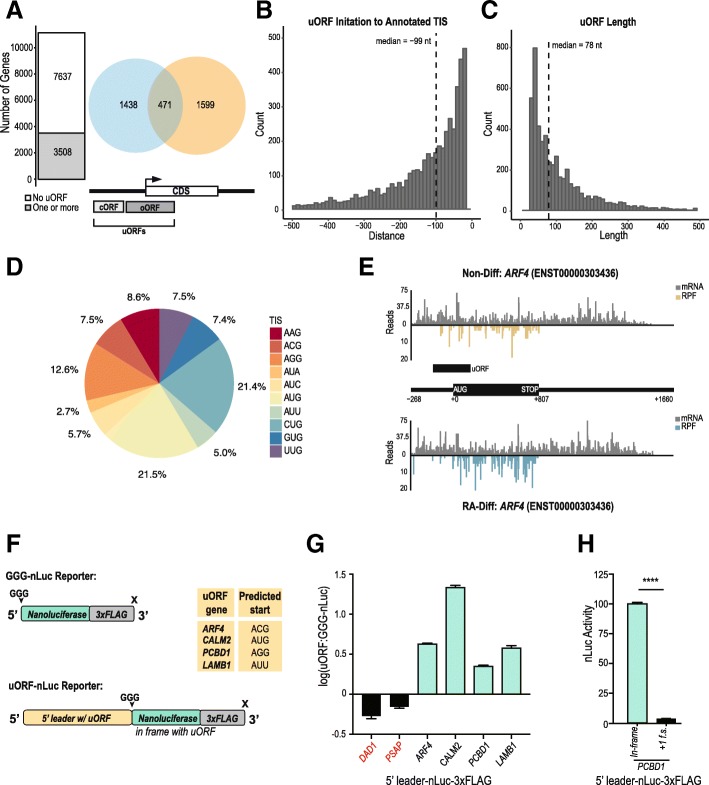
Characterization and validation of predicted uORFs. **a** The number of genes with at least one predicted ORF (bar plot) in the 5′ leader of evaluated protein-coding genes. The number of genes with a predicted ORF terminated upstream in the 5′ leader only (orange), terminated in the CDS only (blue), or with both a predicted upstream- and CDS-terminated ORF (overlap). **b** Distribution of predicted ORF translation initiation position relative to the annotated protein-coding CDS start site. **c** Distribution of predicted ORF lengths. **d** Distribution of uORF translation start sites (TIS). AUG represents all AUGs predicted by SPECtre, or upstream/downstream 30-nt from the SPECtre predicted start site if no intervening stop codon is present. Near-cognate start codons are utilized in the majority of uORFs, while AUG is the single most common start site. **e** Plots show mRNA reads (grey) and RPF counts (cyan/gold) for *ARF4*. The annotated uORF is characterized by the presence of consistent RPF coverage in the 5′ leader. **f** Schematic of the uORF nanoluciferase (nLuc) reporters used in this study. GGG-nLuc serves as a negative control, as its AUG initiation start codon is mutated to a GGG codon. This reporter supports very little translation. A table of the predicted start sites for each uORF reporter. **g** nLuc assays performed in SH-SY5Y cells confirmed expression of these uORFs (teal). 5′ leaders not included in the uORF dataset (black) are below the GGG-nLuc reporter activity and considered not translated. All values are normalized to the GGG-nLuc control performed in parallel, data for individual reporters was collected in triplicate in multiple experiments. Student’s t test, all teal uORFs in panel F) have a *p* value ≤0.0001. Graph represents mean ± S.E.M. **h** Frameshifting the uORF relative to nLuc decreases translation of the reporter. The reporter was cloned so that the nLuc tag was frameshifted (f.s.) out of frame with the predicted uORF and the CDS start site. *n* = 3, Student’s t test, ****p ≤ 0.0001. Graph represents mean ± S.E.M.

We established a translational threshold based on the distribution of scores in known coding genes to establish a minimum SPECtre score needed to classify a region as actively coding with a 5% false discovery rate (FDR) allowed. This results in a set of 3508 transcripts with 4954 unique uORFs (Fig. 2, Additional file 4: Table S4). Of these transcripts, 1599 contained overlapping upstream-initiated ORFs (specified as oORFs), 1438 uORFs fully contained in the 5′ leader (cORFs), and 471 transcripts had two or more uORFs of either of these two categories (Fig. 3a). The median distance of the uORF initiation site from the CDS is 99 nucleotides (Fig. 3b). uORFs have a median length of 78 nucleotides, but can span upwards of 500 nucleotides in length (Fig. 3c). Of note, reads in the 15 nt surrounding the CDS AUG start codon were excluded to avoid errant signal attributed to uORF activation from CDS translation.

Previous work using harringtonine, a drug that stalls ribosomes at initiation sites, revealed a surprising occurrence of near-AUG codons enriched in ribosome peaks [5]. Though near-cognate initiation had been recognized previously, this hinted that there may be a greater number of initiation events at these codons than previously expected [3, 37–41]. When inspecting the translation start site of each SPECtre-identified uORF in our datasets, the majority could not be mapped to an in-frame AUG initiation codon. Translation start sites were plotted to show the relative contribution of each in the final dataset (Fig. 3d). It is important to note that this is the breakdown of start sites within the constraint of our initial parameters, which limited potential start codons to those with a preset identity. AUG initiation sites were accounted for by two different methods: they were either directly identified by SPECtre or factored into the total count if they were present within 30 nucleotides upstream or downstream of the start of the SPECtre signal without an intervening stop site. Due to the high potential translatability of ORFs with AUG start codons, these were all annotated as AUG start sites. This constitutes 21.5% of the initiation sites used. In comparison, we detected 21.4% of uORFs use CUG as their initiation codon, consistent with previous reports [5, 38]. A typical example of RPF and mRNA coverage of a uORF containing transcript is shown for *ARF4,* which encodes a small guanine nucleotide-binding protein important for vesicular trafficking, reveals significant coverage across the uORF in the Non-Diff state (Fig. 3e).

To confirm that SPECtre identified uORFs could support translation we created creating nanoluciferase (nLuc) reporters for a small representative set of genes. For each candidate evaluated, the complete 5′ leader upstream of the start site through the entire predicted coding region of the uORF was placed upstream of an nLuc tag where the AUG start codon was mutated to GGG (Fig. 3f). GGG-nLuc alone, which exhibits very little translational activity in isolation, was used as a negative control [42]. We confirmed SPECtre identified uORFs residing in the 5′ leader of 4 genes: *ARF4*, *CALM2*, *PCBD1*, and *LAMB1*. *ARF4*, *PCBD1,* and *LAMB1* are predicted to utilize near-cognate start sites, while *CALM2* utilizes an AUG (Fig. 3f). Reporters were co-transfected into SH-SY5Y cells with pGL4.13 which encodes firefly luciferase (FFluc) as a transfection control. *DAD1* and *PSAP* served as negative controls, as their 5′ leaders were filtered out early on in our analysis and they exhibited minimal RPF footprints in their 5’UTRs despite robust CDS translation. All 4 of our predicted uORFs showed a significant level of translation above GGG-nLuc (Fig. 3g). One key feature of SPECtre is its ability to discriminate reading frame [14]. To determine if SPECtre correctly predicted the reading frame of uORFs in 5′ leaders, we mutated the reporter for *PCBD1* so that the predicted uORF was out of frame. Placing nLuc out of frame resulted in a significant drop in signal (Fig. 3h), suggesting that SPECtre correctly detected the indicated uORF reading frame.

### uORF/CDS pairs exhibit positively correlated TE shifts with differentiation

**Fig. 4:**
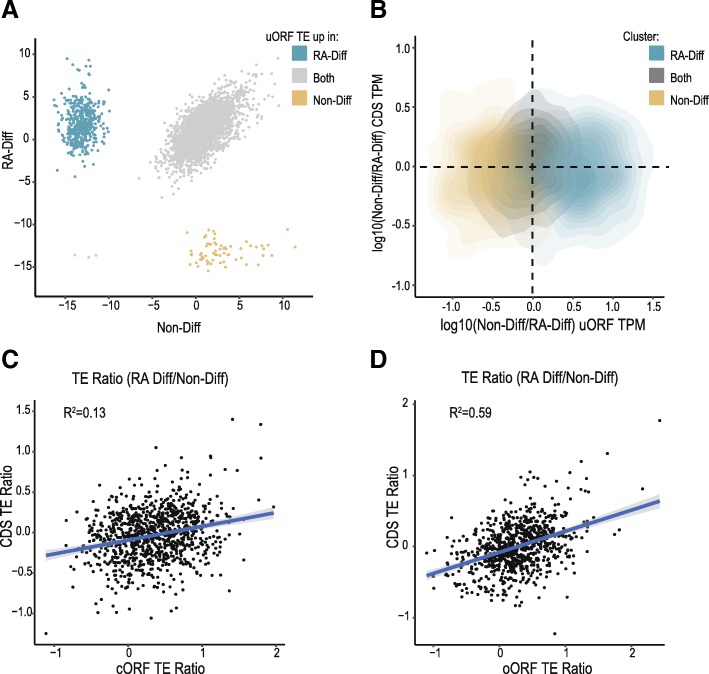
uORFs shift translationally with Retinoic acid induced differentiation. **a** K-means clustering analysis of log_2_(uORF TE) in Non-Diff and RA-Diff cells, reveals differentiation-associated shifts. Three clusters of uORF translation emerge: those that are up-regulated in RA-Diff cells (cyan), up-regulated in Non-Diff cells (gold), and uORFs with no change in translational potential (gray). **b** Clustering in (A) does not correlate with directional CDS changes. Kernel density estimation analysis of changes in TPM over annotated protein-coding CDS as a function of changes in TPM over predicted upstream-initiated ORFs. Cluster identity of predicted ORF changed in translational potential as scored by SPECtre predicted ORFs enriched for translation in RA-Diff cells (cyan), predicted ORFs with enriched translation in Non-Diff cells (gold), and those with static translation across the two conditions (black) are annotated to protein-coding CDS with higher RPF abundance in Non-Diff cells (above horizontal line), and those with higher RPF abundance in RA-Diff cells (below horizontal line). **c** Analysis of transcripts with a cORF reveals a positive correlation of cORF TE and CDS TE. Pearson correlation, R^2^ = 0.13. **d** The same is true for oORFs with a Pearson correlation, R^2^ = 0.59.

To determine whether RA induced differentiation might impact uORF usage, we first performed k-means clustering using the TE of uORFs in the Non-Diff and RA-Diff datasets (Fig. 4a). This revealed that a subset of approximately 14% of predicted uORFs that exhibited a high degree of cell-state specificity (blue and gold) compared to uORFs with consistent TE in both states (grey). A similar relationship was observed for the SPECtre score (Additional file 5: Figure S3A). If these shifts in uORFs translation occur as a means of regulating CDS translation, then we would predict an inverse relationship between these shifts in uORF usage and the TE of their cognate CDS. However, these clusters were not predictive of an inverse directional change in CDS TE (Fig. 4b). Instead, we observed a positive correlation between CDS TE and uORF TE in this dataset (Additional file 5: Figure S3B) that was present regardless of whether we considered all uORF, or cORFs and oORFs individually (Fig. 4c-d). These findings are consistent with a number of previous RP studies [4, 43] and has been interpreted as reflecting enhanced pre-initiation complex loading leads to increases in both uORF and CDS translation, with leaky scanning past the uORF allowing for enhanced initiation at both sites. While this positive relationship does not preclude the potential for specific uORFs to act as repressors, it suggests that global shifts in uORF usage are not driving the majority of changes in CDS TE observed with RA differentiation.

### Even robustly translated uORFs exhibit only modest inhibitory effects on CDS translation

**Fig. 5:**
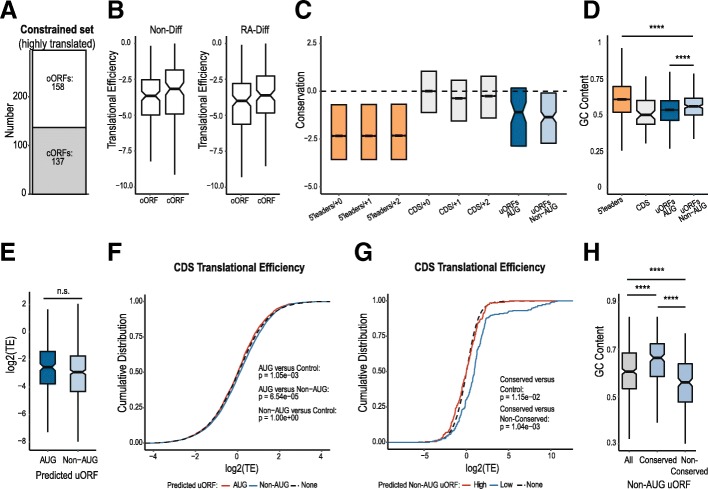
Impact of highly translated uORFS on coding sequence translation. **a** SPECtre identified uORFs were filtered to include only uORFs that have significant coverage in all four Non-Diff and RA-Diff libraries; these are considered highly translated. **b** Average TE values for cORFs and oORFs in the Non-Diff (left) and RA-Diff (right) conditions. **c** Conservation analysis of annotated 5′ leaders in all three reading frames (orange), annotated CDS regions over all three frames (grey), predicted AUG-initiated uORFs (dark blue), and predicted non-AUG uORFs (light blue). **d** Average GC nucleotide content is shown for 5′ leader regions (orange), CDSs (grey), AUG uORFs (dark blue), and non-AUG uORFs (light blue). For oORFs, only the 5′ leader region of the oORF is included. 5′ leaders are significantly more GC rich than both AUG uORFs and non-AUG uORFs, *p* = 5.72e-12 and 1.54e-07, respectively. Non-AUG uORFs are significantly more GC rich than CDSs and AUG uORFs, *p* = 7.92e-18 and 2.16e-06. **e** Average TE for AUG uORFs and non-AUG uORFs reveals no difference between the two subtypes. **f** Empirical cumulative distribution of TE in all CDSs (black) versus CDSs from transcripts with two subsets of uORFs: those with an AUG initiation site (red) and those with a non-AUG initiation site (Blue). **g** Empirical cumulative distribution of TE in all CDSs (black) versus CDSs from transcripts with two subsets of non-AUG uORFs: those in the highest quartile of conservation (Conserved, red) and those in the lowest quartile of conservation (Non-Conserved, Blue). Distributions are significantly different with *p*-values annotated on graph. **h** GC content of non-AUG uORFs grouped by conservation. All versus conserved: *p* = 5.89e-08, all versus non-conserved: *p* = 2.54e-06, conserved versus non-conserved: *p* = 1.62e-18.

As our a priori prediction from biochemical studies was that uORFs should impair cognate CDS translation, we were concerned that inclusion of all uORF containing transcripts identified by SPECtre might mask effects elicited by robustly translated uORFs, which we would predict to have stronger inhibitory effects. We therefore repeated our analysis using a stringent dataset of transcripts including only those transcripts with SPECtre scores indicating highly robust and consistently translated uORFs. This filtering constrained the uORF transcripts to a smaller group of 158 overlapping ORFs (oORFs) and 137 contained ORFs (cORFs) that initiate at either an AUG or near-cognate codon (Fig. 5a). The cORFs and oORFs in this stringent dataset have comparable translational efficiencies (Fig. 5b) and both were more robustly translated than uORFs identified by our initial SPECtre analysis. These uORFs exhibited enhanced conservation at the codon level (comprised of only the 5′ leader region for oORFs) compared to 5′ leader sequences overall (Fig. 5c) and their GC content is lower than 5′ leaders overall, making them more similar to CDS sequences (Fig. 5d) [44, 45]. Somewhat surprisingly, the translational efficiencies of AUG initiated and non-AUG initiated uORFs were comparable in this stringent dataset (Fig. 5e).

Using this constrained set of robustly translated uORFs, we re-evaluated the impact of uORFs on cognate CDS translation. On a population basis, AUG initiated uORFs were nominally associated with less translation from their cognate CDSs, but their effect on a population level was very modest (Fig. 5f), which is consistent with published data [7, 20, 24, 43]. CDS downstream of non-AUG initiated uORFs did not demonstrate any influence on cognate CDS translation (Fig. 5f), although a subtle influence of non-AUG uORFs on cognate CDS translation was observed based on their degree of conservation. For non-AUG uORFs in the top quartile of conservation (conserved), the presence of non-AUG uORFs was associated with CDS repression (Fig. 5g). In contrast, for non-AUG initiated uORFs in the lowest quartile of conservation (non-conserved) overall CDS translation was higher compared to both the total CDS population and the conserved non-AUG uORFs. This difference associated with conservation is not due to differences in the TE of conserved versus non-conserved uORFs, as these were comparable (Fig. 5e). The conserved group exhibited higher GC content, consistent with prior findings demonstrating that 5′ leader secondary structure is a predictor of CDS repression and that these features are conserved (Fig. 5h) [43, 46]. This higher degree of conservation with these particular AUG and non-AUG uORFs is also consistent with previous work exploring the evolutionary constraint on AUG triplets in 5′ leaders of mammalian and yeast transcripts that suggests that uORFs with larger effect are either removed via purifying selection or are heavily conserved [47]. Thus, we can identify both AUG and non-AUG initiated uORFs that inhibit downstream translation. However, the effect sizes are small and they represent a minority (41.4%) of the uORFs detected by ribosome profiling in our datasets.

### Ribosomal transcripts are enriched for uORFs

**Fig. 6:**
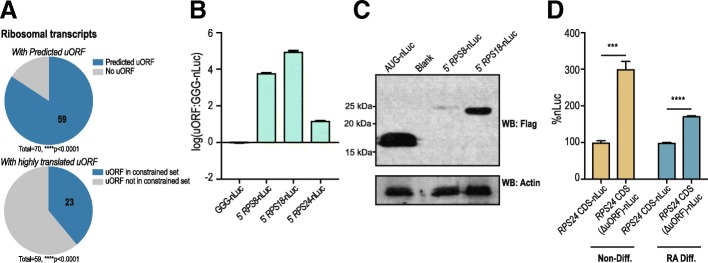
Ribosomal transcripts are enriched in uORF datasets. **a** Top: Chart shows the percent of total actively translated ribosomal protein transcripts (*n* = 70) with a predicted uORF (*n* = 59). Bottom: Chart shows the percent of ribosomal transcripts with uORFs that are in the highly translated dataset (*n* = 23). Ribosomal transcripts are enriched in both sets by Fisher’s exact test, ****p > 0.0001. **b** nLuc assay in SH-SY5Y cells transfected with uORF reporters for ribosomal transcripts: *RPS8* and *RPS18*. **c** Immunoblot of *RPS8* and *RPS18* reporters show an increase in molecular weight relative to AUG-nLuc control, confirming translation initiation within the 5′ leader of these ribosomal transcripts. **d** Removal of the 5′ leader portion of the *RPS24* uORF from nLuc reporters for the *RPS24* CDS increased nLuc signal relative to reporters with the WT *RPS24* 5′ leader.

To explore the potential functional consequences of uORF activity, we performed a GO analysis of transcripts with highly translated uORFs (Additional file 5: Figure S4). These transcripts were significantly enriched in ribonucleoprotein assembly and organization both of which GO categories include the ribosomal proteins. Interestingly, there is a significant enrichment for ribosomal protein transcripts in particular in the full predicted set of uORFs, with 59 uORF containing transcripts, with 19 bearing oORFs and 4 bearing cORFs (Fig. 6a, Additional file 5: Figure S4). Most uORF/CDS pairs in this group of transcripts exhibit a positive correlation in the directionality of changes in translational efficiency in response to differentiation, and in 20 of the 23 ribosomal transcripts, the CDS is more highly translated in the Non-Diff state. We validated the expression of three uORFs from ribosomal protein transcripts: *RPS8, RPS18, and RPS24* by nanoluciferase assay (Fig. 6b). These show robust reporter expression, which was validated by immunoblotting (Fig. 6c). As mutations in the small ribosomal subunit protein *RPS24* cause Diamond-Blackfan Anemia (DBA) [48], we explored this uORF further. In our ribosome profiling data, *RPS24* had a strikingly oppositional relationship to its CDS, with the uORF increasing and the CDS decreasing with differentiation (Additional file 5: Figure S5). The RPS24 uORF overlaps the CDS and is predicted to initiate at an AAG codon. We constructed *RPS24* CDS nLuc reporters with (WT) and without the uORF (ΔuORF) (Fig. 6d). Removing the uORF resulted in a 1.7 fold increase in reporter expression in non-differentiated cells and a 3-fold increase in RPS24 signal in RA-differentiated conditions (Fig. 6d).

### uORFs buffer against differentiation-induced shifts in CDS TE

**Fig. 7:**
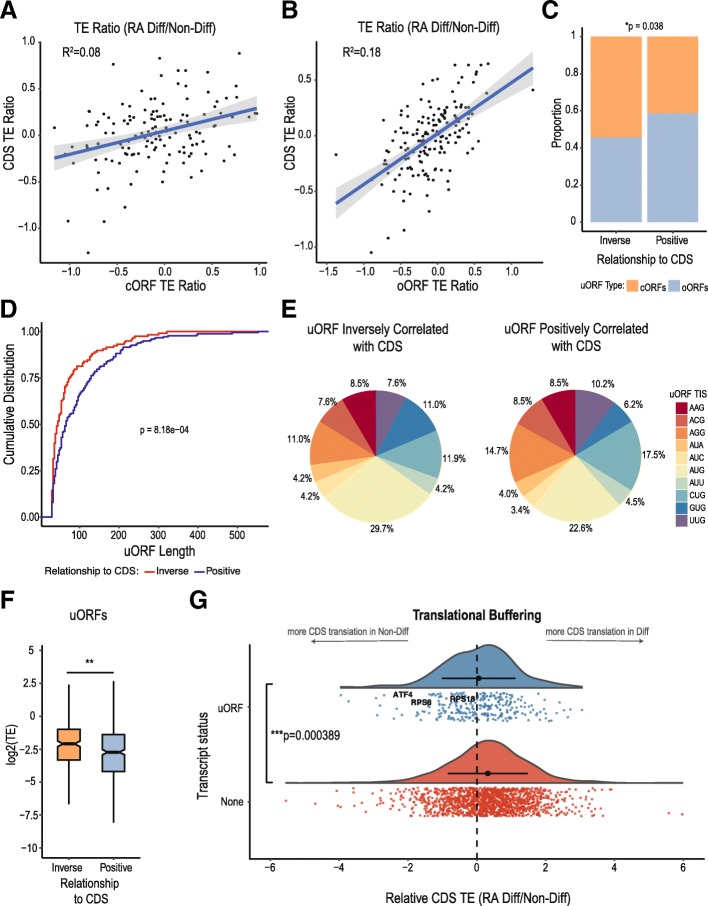
uORFs buffer against differentiation-induced shifts in CDS TE. **a** Analysis of the relationship between cORF and CDS translation (log_10_TE Non-Diff/RA-Diff) for the highly translated dataset reveals that the translational efficiency of these two regions positively correlate in response to RA-Differentiation, R^2^ = 0.08. Regression coefficients were calculated from untransformed TE ratios. **b** This was also seen for oORFs, R^2^ = 0.18. C-E) 40% (118/295, see Additional file 5: Figure S5) of uORF/CDS pairs in the highly translated dataset exhibit TE shifts in the opposite direction for the uORF and the CDS with differentiation (“inverse”), while 60% of uORF/CDS pairs exhibit TE shifts in the same direction with differentiation (“positive”). **c** Relative proportion of “positive” or “inverse” oORFs and cORFs. Chi-square, *p* = 0.038. **d** Distribution of uORFs by length. **e** Start site codon distribution for “positive” or “inverse” uORFs. **f** uORFs with an “inverse” relationship to their associated CDS have a higher average TE than those with a “positive” relationship, *p* = 0.00923. **g** Histograms of log_2_(CDS TE, RA-Diff/TE Non-Diff) for transcripts with a uORF in the highly translated set (uORF) or no uORF (none). ANOVA, *p* = 0.000389.

Given that we observed a modest inhibitory effect of uORFs within our highly translated dataset on CDS translation, we revisited the relationship between shifts in uORF translation and cognate CDS translation with RA induced differentiation in this smaller group of transcripts. However, we again observed an overall positive correlation between conditional translation of uORFs (cORFs and oORFs) and cognate CDS translation between the two conditions (RA-Diff: Non-Diff) with regression coefficients of 0.08 and 0.18 for cORF and oORF transcripts (Fig. 7a-b). A complete list of TE changes with differentiation (TE RA-Diff/TE Non-Diff) in uORF/CDS pairs is shown in Additional file 5: Figure S5. One hundred eighteen transcripts did reliably exhibit inverse relationships between uORF and CDS TE across cell states. We therefore determined if these uORFs had any defining characteristics. A greater percentage of uORFs with this inverse relationship had a predicted AUG initiation site and a higher average TE (Fig. 7e-f). We also observed that there was a greater proportion of cORFs in the inverse group than oORFs (Fig. 7c), and these are shorter in length (Fig. 7d), although these effects are driven predominantly by the co-association of these features with AUG initiated uORFs [7, 49].

As the majority of uORF/CDS pairs exhibited a positive correlation in translational efficiency, we explored potential models whereby uORF activity could influence CDS translation but still exhibit this relationship. One alternative model would be that persistently translated uORFs could limit condition-dependent shifts in CDS translation to act as a homeostatic buffer in the setting of changes in global translational initiation factor activities [50–52]. To test this idea, we analyzed the distribution and relative TE ratio (RA-Diff/Non-Diff) on transcripts with no uORF or with a uORF. The range of TE ratios exhibited significantly increased variance in transcripts without uORFs compared to those with uORFs (Fig. 7g). In addition, transcripts without a uORF showed a significantly lower TE ratio relative to those with a uORF in the highly translated uORF set, suggesting a larger relative increase in TE with differentiation in those transcripts (Fig. 7g). This finding was also observed in for transcripts in the full set of uORFs (Additional file 5: Figure S6). These data support a potential role for uORFs in limiting conditional fluctuations in CDS translation [53].

## Discussion

In this study, we show that application of a spectral coherence algorithm (SPECtre) to RP data and stringent filtering allows for categorization of high confidence uORFs translated in human neuroblastoma cells. The majority of identified uORFs utilize near-cognate start sites, are highly translated, and we were able to experimentally validate a subset of these uORFs using reporter assays. As others have described, AUG initiated uORFs are weakly associated with a repressed CDS [20, 24, 43]. We find a similar relationship occurs with a conserved and highly translated set of non-AUG uORFs, but these effects are quite modest. For a minority (118) of uORF/CDS pairs, exemplified by the ribosomal transcript *RPS24*, we observed that differentiation induced shifts in TE in opposite directions, as would be predicted by the classic model of uORF usage where upregulation of uORF translation leads to a concomitant decrease in translation of the downstream CDS [53, 54]. Yet, for the majority of uORF events, this predicted inverse relationship is not observed. Instead, our data suggests that these uORFs act to buffer state-dependent changes in CDS translation, as state-dependent changes in CDS TE were smaller on uORF containing transcripts. Together, these data support a model whereby uORFs can act as both direct regulators of downstream CDS translation and as homeostatic buffers that constrain changes in translation rates associated with cell state changes.

Past biochemical studies predict that uORFs that do not allow for re-initiation must be bypassed in order to de-repress expression of the downstream CDS, as has been observed on *GCN4* in yeast in a process termed delayed reinitiation [54–58]. There are two determining cORFs upstream of the *GCN4* CDS, with the first showing persistent translation, and the second acting under normal conditions to preclude the scanning ribosome from reaching the CDS with a ternary complex for initiation [54]. In delayed reinitiation, under conditions of starvation, the second uORF is bypassed due to limiting ternary complexes which allows scanning ribosomes to continue on and load ternary complexes prior to reaching the CDS [9]. Hence, GCN4 protein is upregulated in response to cellular stress and eIF2α phosphorylation.

This traditional model of uORF mediated regulation is not borne out in our own data for the majority of uORFs. Instead, our data is more consistent with previous ribosome profiling reports in yeast and mammalian cells suggesting a positive relationship between uORF translation and CDS translation [4, 43, 59]. One proposed mechanism for this positive correlation is the low efficiency of most uORF translation and a reliance on whole transcript activity to increase in order to detect such rare events. The scanning model of translation initiation predicts that if uORF usage at suboptimal codons is an inefficient event, that it would increase as the number of loaded preinitiation complexes increase on a transcript [6, 39]. Thus, cap binding and initiation of scanning would be the rate limiting steps for both the uORF and CDS translation. Our data are largely consistent with this model. Even after a priori filtering and additional constraining of our dataset, we still observe a clear positive correlation of shifts in translational efficiency (TE RA-Diff/TE Non-Diff) for uORF/CDS pairs, and this positive relationship is maintained even for overlapping uORFs which should preclude CDS re-initiation.

If uORFs are not strongly repressive of CDS translation, then what purpose might they serve? One hint comes from earlier studies that used ribosome profiling on cells treated with stressors such as arsenite and tunicamycin, and showed that CDSs on transcripts with uORFs either increase in TE or remained resistant relative to global decreases in translation [50, 52]. This included transcripts with known uORFs as well as transcripts that were later found to support uORF translation. Differentiation was associated with an increase in CDS TE on transcripts without a uORF but not on transcripts with a uORF, which showed significantly smaller shifts with differentiation overall. These data are consistent with uORFs acting not as on/off switches to control CDS translation, but instead as buffers to changes in TE of their associated CDS that occur with cell state shifts [60–62].

We identified ribosomal protein encoding transcripts as being particularly enriched for uORFs. A decrease in translation complex-related transcripts was previously detailed in neuronal differentiation and attributed to a decrease in mTORC1 activity [23]. However, uORFs are another potential candidate for the regulation of this class of proteins. Most ribosomal transcripts, including all transcripts in this subset, have a 5′ terminal oligopyrimidine (5′ TOP) motif which implements translational control mediated by stress and the mTOR signaling pathway [63]. As the majority of uORF/CDS pairs on ribosomal transcripts were co-repressed with retinoic acid induced differentiation, these 5′ TOP motifs may exert similar effects on both the uORF and CDS translation. An important exception to this relationship was observed for the small ribosomal subunit transcript *RPS24,* where a robustly translated non-AUG initiated uORF exhibited a strong inverse relationship to the *RPS24* CDS with differentiation. Mutations in *RPS24,* which lower the levels of this small ribosomal subunit protein, cause Diamond-Blackfan Anemia (DBA) [48]. Levels of this protein can also affect the abundance of another key ribosomal protein mutated in DBA, *RPS19*, and its altered production has the potential to disturb ribosomal stoichiometry necessary for proper translation [48, 64–66]. Thus, this uORF has the potential to contribute to *RPS24* expression in patient cells.

## Conclusions

We utilized ribosomal profiling to detect uORFs in a human neuroblastoma cell based model and defined how their expression changed with cellular differentiation. This work provides insights into the dynamic relationships and potential regulatory functions of uORF/CDS pairs in a model of neuronal differentiation and suggest a specific role for uORFs in constraining gene expression changes that occur with shifts in cell state. Future studies will be needed to define how individual uORF/CDS relationships are regulated and how they influence specific steps in neuronal differentiation.

## Methods

### SH-SY5Y cell maintenance and differentiation

SH-SY5Y cells were acquired from ATCC (CRL-2266). Cells were grown in DMEM:F12 media (Invitrogen) supplemented with 10% FBS, .01 mg mL^− 1^ Gentamicin and .25μg mL^− 1^ Amphoreticin B. Cells were plated on 150 mm plates that were either coated with .1 mg/mL poly-D lysine (Millipore) for differentiation or uncoated. Cells were allowed to propagate to 80% confluency for 1–2 days prior to lysing for ribosome profiling or induction of differentiation. SH-SY5Y cells were differentiated for 6 days in 10 μM retinoic acid (all-trans, Sigma), with media changed every 24 h.

### Construction of the ribosome profiling libraries

Ribosome profiling libraries were prepared as in Ingolia et al., 2010 and Ingolia et al., 2012. Cells were washed with ice cold PBS with CHX at 100μg mL^− 1^. Plates were immediately flash frozen in liquid nitrogen, moved to dry ice, and lysed (in the presence of CHX) to prevent ribosome loading and runoff. Additional lysates were processed in parallel for poly(A) mRNA purification and mRNA-sequencing library preparation. Polysomes were isolated from the ribosome footprinting lysates on a 1 M sucrose cushion with high speed centrifugation using a 70.1Ti rotor (Beckman) at 55,000 r.p.m. for 4 h at 4 °C. rRNA was eliminated prior to linker ligation using Ribo-Zero Gold rRNA Removal Kit (Illumina). Ribosome Profiling libraries were barcoded and multiplexed with 2–4 libraries per lane, and sequenced on a HiSeq 2000 (Illumina) using 50 cycles of single end reads. mRNA libraries were multiplexed on a single lane. All sequencing was conducted at the University of Michigan DNA Sequencing Core.

### Plasmid construction

pcDNA 3.1 plasmid was modified to encode NanoLuc and GGG-NanoLuc as previously published [42]. gBlocks® (IDT) were ordered of the 5′ leader sequence to the last codon before the in-frame stop of selected genes flanked by restriction sites. These were restriction cloned upstream of GGG-nLuc using PacI and XhoI (NEB) with 12 nucleotides between the start of the 5′ leader and the T7 promoter sequence to reduce spurious initiation in sequences specific to the plasmid. Frameshifts were accomplished by PCR cloning with primers that inserted one or two nucleotides between the uORF and the nanoluciferase sequence. PCR products were cloned in place of nanoluciferase in the original uORF plasmid using XbaI and SacII (NEB). Restriction digest and Sanger Sequencing were used to confirm plasmid sequence.

### SH-SY5Y transfection and Nanoluciferase assay

SH-SY5Y cells were seeded on 6-well culture plates at 3 × 10^5^ cells per well. 24 h post seeding, each well was transfected using 7.5 μL FUGENE HD (Promega) and 1.25 μg nanoluciferase reporter plasmid along with 1.25 μg pGL4.13 (internal transfection control that encodes firefly luciferase [FFluc]) in 300 μL of OptiMEM (Invitrogen). Transfections of differentiated cells were performed on day 5 in RA supplemented media. Cultures were allowed to grow for 24 h after transfection. Cells were lysed in 250 μL Glo Lysis Buffer (Promega) for 5 min at room temperature. 50 μL lysate was mixed with 50 μL prepared Nano-GLO or ONE-Glo (Promega) for 2 min, and bioluminescence was detected using a GloMax® 96 Microplate Luminometer. Nanoluciferase signal was normalized to FFluc signal in each sample. pcDNA 3.1 encoding nLuc the AUG start codon mutated to a GGG (GGG-nanoLuc) was run in parallel with each experimental nLuc plasmid and subjected to both conditions to serve as a control for normalization.

### Immunocytochemistry and microscopy

Cells were fixed at 37 °C with 4% PFA/4% sucrose in PBS with 1 mM MgCl_2_ and .1 mM CaCl_2_ (PBS-MC), permeabilized for 5 min in .1% Triton-X in PBS-MC, and blocked for 1 h with 5% bovine serum albumin in PBS-MC. Cells were incubated in blocking buffer and primary antibodies against β-actin (Santa Cruz Biotechnology, cat# sc-130,656, 1:1000) and neurofilament (Abcam, Ab8135, 1:1000) for 1 h at room temperature. Following 3 × 10 minute washes in PBS-MC, cells were incubated in PBS-MC with Alexa Flour 488 conjugated goat anti-rabbit IgG and Alexa Flour 635 conjugated goat anti-mouse IgG to achieve secondary detection (Thermo Fisher, 1:1000). Cells were washed again, and placed in ProLong™ Gold antifade reagent with DAPI (Invitrogen).

Imaging was performed on an inverted Olympus FV1000 laser-scanning confocal microscope using a 40x objective. Acquisition parameters were identical for each condition and optimized to eliminate signal bleed-through between channels. Images were converted to average-intensity z-projections in ImageJ. Cytoplasmic β-actin was quantified by averaging the integrated density corrected for background signal of the cells in each condition. The length of one main neurofilament-labeled primary neurite per cell was determined in ImageJ and converted from pixels to μm, and averaged for each condition.

### Western blotting

Cells were maintained as described above. Cells were washed 2X in PBS, and RIPA buffer was added to a single well of a 12-well dish either at 80% confluency or after 6 days of retinoic acid differentiation. Cells were agitated for 40 min at 4 °C to ensure complete lysis. Lysates were clarified by centrifugation, and the supernatant was mixed with reducing SDS sample buffer and boiled for 5 min at 90 °C. Equal amounts of lysate were loaded on an SDS-PAGE gel and subsequent western blotting was carried out with primary antibodies against FMRP (1:1000, cat# 6B8, BioLegend), GAPDH (1:1000, cat# sc-32,233, Santa Cruz Biotechnology), total eIF2α (1:1000, cat# 9722, Cell Signaling Technology), phospho-eIF2α (1:500, cat#: 44-728G, Invitrogen) or E7 Tubulin (1:1000, DSHB)—in 5% (wt/vol) non-fat dry milk in TBS-T (NFDM). An HRP conjugated goat antibody to mouse IgG or to rabbit IgG was used for secondary detection (1:5000, Jackson ImmunoResearch Laboratories) in 5% NFDM. SuperSignal™ West Femto Maximum Sensitivity Substrate (Thermo Scientific) was used for HRP detection of phospho-eIF2α levels. Western Lightening® Plus-ECL (PerkinElmer, Inc.) was usedfor all other antibody detection.

### Alignment to the human genome and transcriptome (GRCh38 Ensembl version 78)

Ribosome profiling and mRNA-Seq reads were trimmed of adapters, and then by quality using *fasqt-mcf* from the *ea-utils* package (Aronesty, 2011). Ribosome profiling and mRNA-Seq reads in FASTQ format were trimmed based on quality if four consecutive nucleotides were observed with Phred scores of 10 or below. The minimum read length required after trimming was 25 nucleotides.

Trimmed sequences were then aligned to a ribosomal RNA sequence index using Bowtie v1.1.2 (Langmead, *et. al.,* 2009) to deplete them of contaminant sequences. Alignment to the rRNA sequence contaminant index was performed using the following parameters: seed alignment length of 22 nucleotides, no mismatches in the seed alignment were allowed, with the unmapped reads written to a separate FASTQ file.

bowtie -l 22 -n 0 -S --un /path/to/depleted_reads.fq \

/path/to/rRNA_index \

/path/to/trimmed_reads.fq

Ribosome profiling and mRNA-Seq reads depleted of rRNA contaminant sequences were aligned to the human genome and transcriptome (Ensembl, version 78) using TopHat v.2.0.10 (Trapnell*, et. al.,* 2009). The trimmed and rRNA-deplete reads were aligned to the human genome and transcriptome with the parameters specifying standard Illumina reads, with un-gapped Bowtie 1 alignment (using a seed alignment length of 22 nucleotides, with no mismatches in the seed alignment allowed), to annotated junctions only, using Solexa quality scores:

tophat2 -p 4 –bowtie1 \

–-no-novel-juncs \

--library-type fr-unstranded \

--solexa-quals \

-G /path/to/ensemble.gtf \

/path/to/bowtie_index \

/path/to/depleted_reads.fq

### Sequence alignment quality filtering and merging

Ribosome profiling and mRNA-Seq reads aligned to the human genome and transcriptome by TopHat2 were output to BAM format, and then sorted by chromosomal coordinate. Reads were then filtered by mapping quality using SAMtools (Li, *et. al.,* 2009); read alignments were required to have minimum mapping quality of 10, or higher, to be retained for subsequent analyses. Unique read group identifiers were assigned to each technical and biological replicate, and then the alignments were merged by technical replicates and subsequently as biological replicates using Picard (http://broadinstitute.github.io/picard/).

### Metagene profile generation and alignment offset calculation

For counting reads over transcript isoforms, metagene profiles were generated from the Ensembl (version 78) transcript annotation database using Plastid (Dunn*, et. al.*, 2016). A- and P-site offsets for harringtonine and cycloheximide ribosome profiling reads, respectively, were determined by pooling all reads that overlapped canonical AUG translation initiation start sites from annotated protein-coding genes. The most common (mode) distance from the 5′ ends of reads of a given length to the position of the AUG in those reads was accepted as the A- or P-site offset distance.

### Calculation of transcript abundance

Read counts over each transcript isoform, or region (5’UTR, CDS, and 3’UTR), were normalized by length, summed, and reported as transcripts per million (TPM) as described previously (Li, *et. al.,* 2011). At the time of analysis, Cufflinks (Trapnell, *et. al.,* 2010) was required for initial transcript quality control, and was run with the following parameters:

cufflinks -p 8 -o /path/to/output \

-G /path/to/ensemble.gtf \

/path/to/tophat/alignments

### SPECtre analysis of transcripts in non-differentiated and RA-differentiated libraries

SPECtre profiling (Chun, *et. al.,* 2016) measures the strength of the tri-nucleotide periodicity inherent to the alignment of ribosome protected fragments to protein-coding genes in a reference transcriptome. SPECtre analysis was applied in two stages: 1) to score the translational potential of annotated transcripts to build a background protein-coding reference model, and 2) to score the translational potential of predicted upstream-initiation ORFs. In this way, the translational potential of predicted upstream and overlapping ORFs are score against a background model of translation derived from annotated protein-coding transcripts. Annotated protein-coding transcripts were profiled by SPECtre using the following parameters:

python /path/to/SPECtre.py \

--input /path/to/tophat/alignments \

--output /path/to/output \

--log /path/to/logfile \

--gtf /path/to/ensemble.gtf \

--fpkm /path/to/cufflinks/isoforms.fpkm_tracking \

--len 30 \

--fdr 0.05

--min 3.0 \

--nt 8 \

--type mean \

--target <chromosome_id>

Where the minimum FPKM required for a transcript to be considered as translated for generation of the background model was specified as 3.0, and the length of the sliding SPECtre windows was set to encompass 30 nucleotides. The SPECtre score for a transcripts was defined as the mean of the scores over these sliding windows, and a 5% false discovery rate was established to set the minimum SPECtre translational score threshold. In addition, SPECtre profiling was split by chromosome to speed computation, and the results were merged afterwards using a custom Python script. Finally, prior to analysis of predicted upstream-initiated ORFs by SPECtre profiling, the minimum SPECtre translational threshold was re-calculated using TPM instead of FPKM using a minimum cutoff of 10 transcripts per million.

### Computational prediction of upstream-initiated open reading frames

Open reading frames were computationally predicted from annotated 5’UTR sequences (Ensembl, version 78) using AUG, and near-cognate non-AUG translation initiation site sequences. Open reading frame sequences were generated based on these predicted initiation site sequences and read through to the first in-frame termination codon encountered in the annotated CDS. These predicted ORFs were then used to generate coordinates over which they would be profiled and scored by SPECtre. Identical parameters to the annotated transcript SPECtre analysis were employed for consistency across analyses:

python /path/to/SPECtre-uORFs.py \

--input /path/to/alignments \

--output /path/to/output

--results /path/to/spectre/transcript_results \

--log /path/to/logfile \

--fpkm /path/to/cufflinks/isoforms.fpkm_tracking \

--len 30

--fdr 0.05

--min 3.0 \

--nt 8 \

--type mean \

--target <chromosome_id>

Three alternative inputs are required for the SPECtre analysis of predicted ORFs: 1) the annotated transcript GTF database was not required and removed, 2) the results of the annotated transcript analysis, and 3) a genomic sequence file in FASTA format. The results of the annotated transcript analysis were used to identify the set of transcripts from which to predict upstream-initiated ORFs, and the FASTA sequence file was used to generated the ORF sequences for output.

### Supplemental annotation of non-AUG translation initiation sites

Upstream sequences of predicted non-AUG translation initiation sites were examined for possible in-frame AUG initiation start sites; 5’UTR sequences of predicted non-AUG sites were extracted, and then searched for the presence of in-frame AUG sites. These non-AUG sites were then re-annotated according to the proximity of upstream AUG initiation sites: those with an in-frame AUG site within 30 nucleotides of the predicted TIS, and those with an in-frame AUG site in-frame, but beyond 30 nucleotides upstream of the predicted site.

### Kernel density estimation of differential uORF translation on CDS translational efficiency

To further differentiate those uORFs with differential translation and identify those that contribute to the regulation of downstream CDS, the log-change in predicted ORF TPM was compared against the log-change in downstream CDS TPM across the conditions. The differential translational identity of each predicted ORF was retained from the SPECtre clustering analysis, and kernel density estimation was performed using R.

### Heuristic filtering of predicted uORFs

Candidate uORFs were filtered based on a series of heuristic criteria, including: 1) the removal of predicted uORFs with no RPF coverage in the 5’UTR of the transcript, 2) the removal of uORFs predicted to initiate within 15-nt of the annotated CDS start site, and 3) the removal of predicted uORFs without matching mRNA-Seq coverage in the 5’UTR of the transcript. Following these initial minimal coverage filters, the candidate uORFs were further stratified by quality of coverage. First, identical uORF isoforms in overlapping transcripts within the same protein-coding gene annotation set were merged into a single candidate. Next, overlapping uORF candidates were prioritized by the extent of RPF coverage to their 5′ end, as well as by overall coverage. Finally, any remaining overlapping uORF candidates were prioritized by the magnitude of their calculated SPECtre score, with higher scored candidates preferred. R code for the functions to execute the heuristic filtering of uORF candidates is replicated in Supplemental Methods.

### Calculation of translational efficiency

Ribosome profiling or mRNA-Seq reads were counted over each region (5’UTR, CDS, and 3’UTR), transcript, or uORF and then normalized to length and library size as transcripts per million [67]. To calculate translational efficiency over a region, transcript or uORF, ribosome profiling TPM in each biological replicate across each condition was quantile normalized (Amaratunga*, et. al.,* 2001) and then divided by the quantile normalized TPM in mRNA-Seq. Read and RPF counts from mRNA-Seq and ribosome profiling libraries does not include those that overlap the 5’UTR and 3’UTR. Furthermore, to limit the boundary effects due to translation initiation and termination, RPF and read counts do not include those reads whose A- or P-site adjusted position for harringtonine and cycloheximide libraries, respectively, overlap the first or last 15 nucleotides of an annotated CDS.

### Differential expression analysis and gene set enrichment testing in mRNA-Seq

As described previously, the read abundance over annotated protein-coding transcripts was calculated as TPM, then quantile normalized across conditions using the preprocessCore package (Bolstad, 2016) in R (R Core Team, 2017), and then ranked. The change in rank for each gene was calculated across the non-differentiated and RA-differentiated conditions, and the significance of the up- or down-regulation of these rang-changes across conditions was classified using an extreme outlier cutoff [68]. Functional characterization of these significantly rank-changed genes across the non-differentiated and RA-differentiated conditions was analyzed using the goseq package [69] in R, and corrected for multiple testing using Benjami-Hochberg adjusted *p*-values.

### Differential translation analysis and gene set enrichment testing in ribosome profiling

Ribosome profiling read fragments were A- or P-site adjusted, and then counted over annotated protein-coding CDS regions in each biological replicate using the metagene profiles generated by Plastid [70]. As described previously, ribosome-protected fragments with A- or P-site adjusted positions that overlapped the first or last 15 nucleotides of the boundaries defined by the annotated CDS region were masked from the analysis. DESeq2 [71] was used to identify those genes with differential translation across the two states of cellular differentiation. Genes were annotated as significantly up- or down-regulated using a Benjamini-Hochberg adjusted p-value cutoff of 0.1, and fold-change in counts greater than 1, or less than 1, respectively. Functional characterization of these significantly up- and down-regulated genes was analyzed by goseq using parameters specified previously.

### Differential translational efficiency and gene set enrichment testing in ribosome profiling

For each biological replicate, ribosome profiling read fragments were A- or P-site adjusted, and then counted over annotated protein-coding CDS regions using the metagene profiles generated by Plastid. As above, read counts over the first and last 15 nucleotides of protein-coding CDS regions were masked for subsequent analyses. In addition, mRNA-Seq read counts were extracted from each condition, with the proximal and terminal 15 nucleotide ends of the CDS masked for consistency with the RPF counts. The DESeq2 wrapper for differential translational efficiency analysis, Riborex [72], was used to identify those genes with significant changes in translational efficiency. Genes were annotated as significantly up- or down-regulated using a Benjamini-Hochberg adjusted p-value cutoff of 0.1, and absolute fold-change of 1. Functional characterization of the sets of genes enriched in each condition by translational efficiency was analyzed by goseq using parameters described previously.

### Conservation analysis

To assess the conservation of the various regions, transcripts and uORFs, the PhyloCSF scores [73] over each target region was extracted. For uORFs, the PhyloCSF score was extracted according to its predicted phase. In order to de-convolute the contribution of regional conservation due to overlap with annotated CDS regions, predicted uORFs that did not initiate and terminate wholly upstream of a CDS were also scored according to the subset of their coordinates defined by the 5’UTR alone. The mean PhyloCSF over each of these regions and uORFs was calculated, and then mean-shifted to the canonical (+ 0) reading frame of the annotated CDS for comparison.

### GC nucleotide content analysis

Similar to the conservation scores, the ratio of GC nucleotide content in each reading frame of 5’UTRs, and CDS. GC content over the predicted phase of each uORF was calculated, with the 5’UTR overlapping region of CDS-terminated uORFs deconvoluted from the region overlapping the CDS as described above.

### Cluster analysis of differential uORF translation by SPECtre score

In order to identify subsets of uORFs with differential translation in one state of cell differentiation compared to the other, the SPECtre score for each predicted uORF was calculated (described in Supplemental Materials and Methods). The SPECtre score of each predicted uORF was classified by k-means clustering in R to define sets of uORFs with differential translation in one of the conditions, and those with no difference in translational potential between the two conditions.

### Additional filtering of candidate uORFs

Additional replicate-based filtering was applied to the set of predicted uORFs to identify a set of highly confident candidates. As a form of internal validation, a predicted uORF was required to meet a minimal translational threshold in at least one of the biological replicate samples across both conditions. This threshold was determined on a conditional basis dependent on the 5% FDR cutoff required for translational activity according to the distribution of SPECtre scores in protein-coding genes.

## Additional files

Additional file 1: **Table S1.** Reads mapped by Bowtie. This table outlines the breakdown of sequencing reads for each individual replicate. (XLSX 12 kb)

Additional file 2: **Table S2.** Analysis of mRNA sequencing and Ribosome Profiling. All mRNA, RPF, and TE changes are listed in this table for all transcripts. (XLSX 2581 kb)

Additional file 3: **Table S3.** Full GoSeq analysis. Results from gene ontology analysis by mRNA, RPF, and TE measures. (XLSX 10 kb)

Additional file 4: **Table S4.** Full dataset of uORFs. All uORFs detected by SPECtre are listed and scored alongside their associated CDS. (XLSX 2835 kb)

Additional file 5: **Figure S1.** Gene sets with significantly downregulated or upregulated mRNA transcripts in RA-Diff cells. Genes sets for Biological Process are shown in **(A)** and **(B)**, sets for Molecular Function are shown in **(C)** and **(D)**, and sets for Cellular Compartment are shown in **(E**) and (**F**). The top five groups with significant change using a multiple testing corrected *p*-value cutoff of 0.05 (vertical line) are shown on the graph. **Figure S2.** Gene sets with significantly downregulated or upregulated Translational Efficiency in RA-Diff cells. Genes sets for Molecular Function are shown in (**A**) and (**B**), and sets Cellular Compartment are shown in (**C**) and (**D**). The top five groups with significant change using a multiple testing corrected p-value cutoff of 0.05 (vertical line) are shown on the graph. **E**) Plot shows normalized mRNA reads (grey) and RPF (cyan/gold) over the 5’leader (thin line, left), and CDS (thick line, middle). *DAD1* is an example of a transcript with an increase in both mRNA reads and RPFs, leading to no overall change in TE. **Figure S3. A**) K-means clustering analysis of log_2_(uORF SPECtre Score) in Non-Diff and RA-Diff cells. Three clusters emerge: uORFs with an up-regulated TE in RA-Diff cells (cyan), uORFs with an up-regulated TE in Non-Diff cells (gold), and uORFs with no change in TE (gray). **B**) Analysis of the full uORF-containing transcript set reveals a positive correlation of uORF TE and CDS TE. Pearson correlation, r = 0.41. **Figure S4.** cORF and oORF transcripts are graphed separately to show the direction of CDS and uORF TE shifts (RA-Diff/Non-Diff). Significant TE changes are represented as colors specified by the heat-map. ***denotes ribosomal transcripts. **Figure S5.** Histograms of log_2_(CDS TE, RA-Diff/TE Non-Diff) for transcripts with a uORF in the full set (uORF) or no uORF (none). ANOVA, *p* = 0.0322. **Figure S6 (PDF 6057 kb)**

## Abbreviations

CDS: Coding sequence
cORF: Contained upstream open reading frame
nLuc: Nanoluciferase
Non-Diff: Non-differentiated
oORF: Overlapping upstream open reading frame
RA: Diff- retinoic acid differentiated
RP: Ribosome profiling
TE: Translational efficiency
uORF: Upstream open reading frame
