# Supplementary material for: Translation of upstream open reading frames in a model of neuronal differentiation
Source: BMC Genomics. 2019 May 20;20:391. doi: 10.1186/s12864-019-5775-1 (PMC6528255; doi:10.1186/s12864-019-5775-1)
Supplement: Supplementary file 5 — Figure S1. Gene sets with significantly downregulated or upregulated mRNA transcripts in RA-Diff cells. Genes sets for Biological Process are shown in (A) and (B), sets for Molecular Function are shown in (C) and (D), and sets for Cellular Compartment are shown in (E) and (F). The top five groups with significant change using a multiple testing corrected p-value cutoff of 0.05 (vertical line) are shown on the graph. Figure S2. Gene sets with significantly downregulated or upregulated Translational Efficiency in RA-Diff cells. Genes sets for Molecular Function are shown in (A) and (B), and sets Cellular Compartment are shown in (C) and (D). The top five groups with significant change using a multiple testing corrected p-value cutoff of 0.05 (vertical line) are shown on the graph. E) Plot shows normalized mRNA reads (grey) and RPF (cyan/gold) over the 5’leader (thin line, left), and CDS (thick line, middle). DAD1 is an example of a transcript with an increase in both mRNA reads and RPFs, leading to no overall change in TE. Figure S3. A) K-means clustering analysis of log2(uORF SPECtre Score) in Non-Diff and RA-Diff cells. Three clusters emerge: uORFs with an up-regulated TE in RA-Diff cells (cyan), uORFs with an up-regulated TE in Non-Diff cells (gold), and uORFs with no change in TE (gray). B) Analysis of the full uORF-containing transcript set reveals a positive correlation of uORF TE and CDS TE. Pearson correlation, r = 0.41. Figure S4. cORF and oORF transcripts are graphed separately to show the direction of CDS and uORF TE shifts (RA-Diff/Non-Diff). Significant TE changes are represented as colors specified by the heat-map. ***denotes ribosomal transcripts. Figure S5. Histograms of log2(CDS TE, RA-Diff/TE Non-Diff) for transcripts with a uORF in the full set (uORF) or no uORF (none). ANOVA, p = 0.0322. Figure S6 (PDF 6057 kb) [file 12864_2019_5775_MOESM5_ESM.pdf]

Figure S1

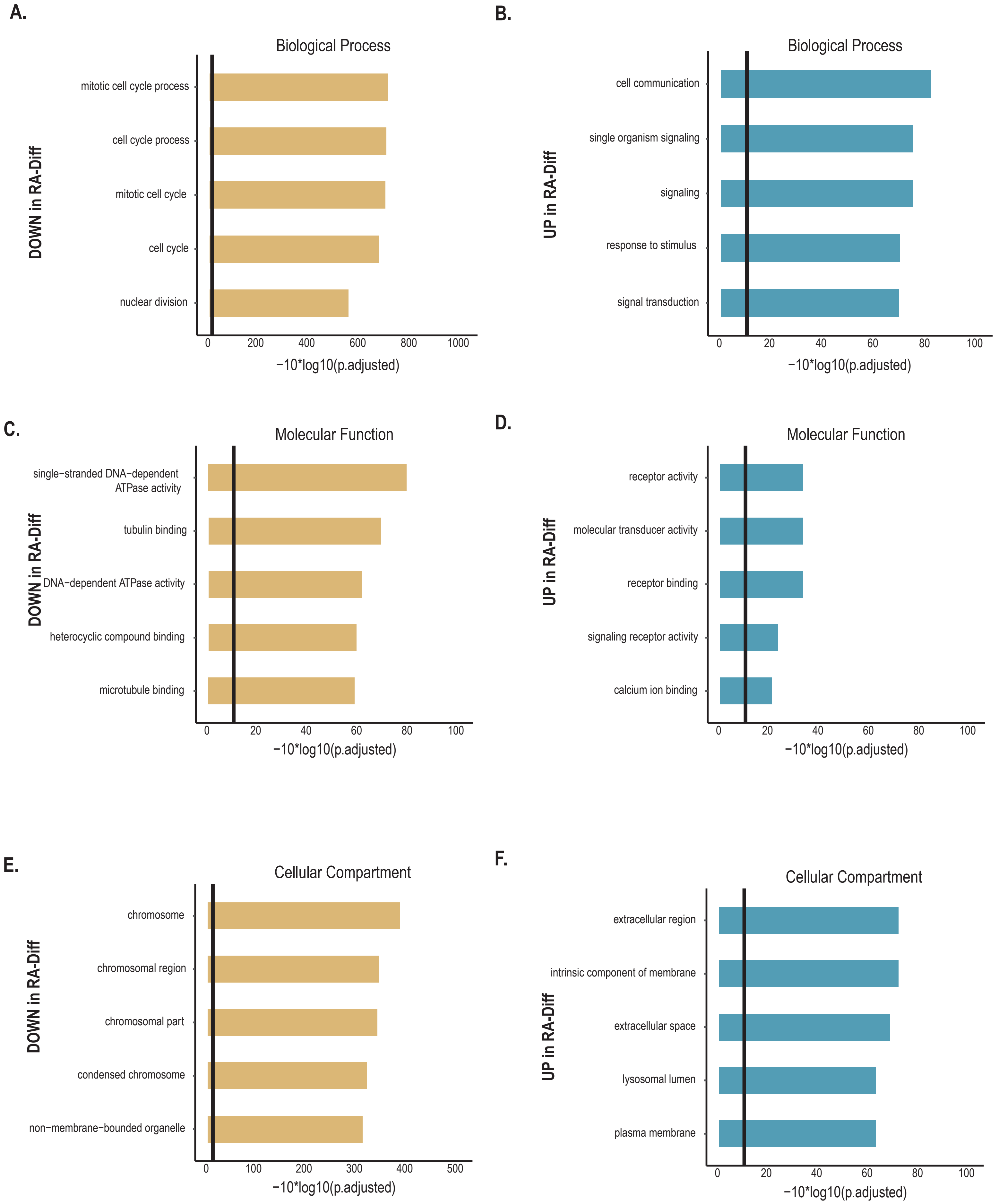
A.
Biological Process
DOWN in RA-Diff
mitotic cell cycle process
cell cycle process
mitotic cell cycle
cell cycle
nuclear division
0
200
400
600
800
1000
−10*log10(p.adjusted)
B.
Biological Process
UP in RA-Diff
cell communication
single organism signaling
signaling
response to stimulus
signal transduction
0
20
40
60
80
100
−10*log10(p.adjusted)
C.
Molecular Function
DOWN in RA-Diff
single−stranded DNA−dependent ATPase activity
tubulin binding
DNA−dependent ATPase activity
heterocyclic compound binding
microtubule binding
0
20
40
60
80
100
−10*log10(p.adjusted)
D.
Molecular Function
UP in RA-Diff
receptor activity
molecular transducer activity
receptor binding
signaling receptor activity
calcium ion binding
0
20
40
60
80
100
−10*log10(p.adjusted)
E.
Cellular Compartment
DOWN in RA-Diff
chromosome
chromosomal region
chromosomal part
condensed chromosome
non−membrane−bounded organelle
0
100
200
300
400
500
−10*log10(p.adjusted)
F.
Cellular Compartment
UP in RA-Diff
extracellular region
intrinsic component of membrane
extracellular space
lysosomal lumen
plasma membrane
0
20
40
60
80
100
−10*log10(p.adjusted)

Figure S2

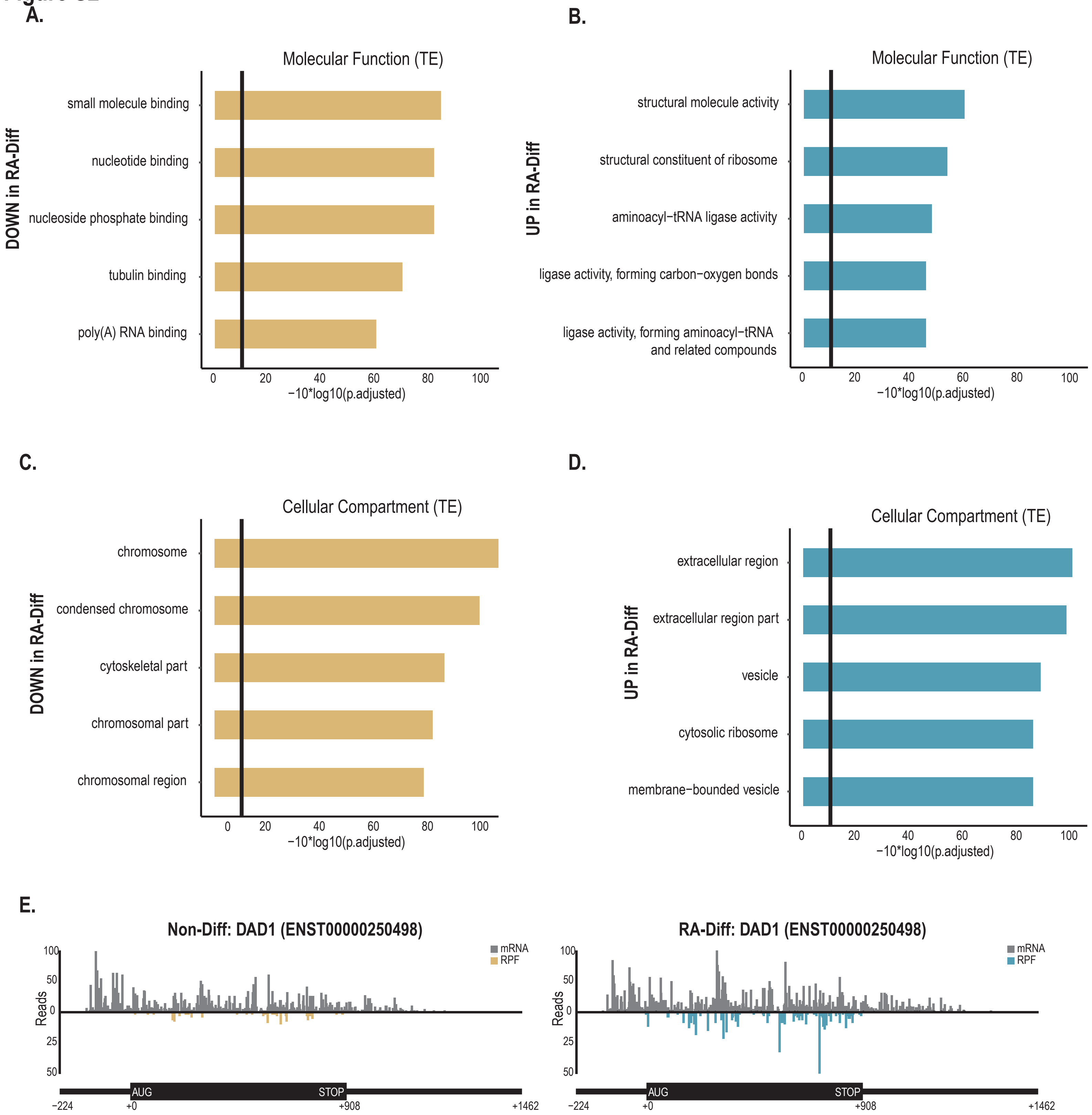
A.
Molecular Function (TE)
DOWN in RA-Diff
small molecule binding
nucleotide binding
nucleoside phosphate binding
tubulin binding
poly(A) RNA binding
0
20
40
60
80
100
−10*log10(p.adjusted)
B.
Molecular Function (TE)
UP in RA-Diff
structural molecule activity
structural constituent of ribosome
aminoacyl−tRNA ligase activity
ligase activity, forming carbon−oxygen bonds
ligase activity, forming aminoacyl−tRNA and related compounds
−10*log10(p.adjusted)
C.
Cellular Compartment (TE)
DOWN in RA-Diff
chromosome
condensed chromosome
cytoskeletal part
chromosomal part
chromosomal region
−10*log10(p.adjusted)
D.
Cellular Compartment (TE)
UP in RA-Diff
extracellular region
extracellular region part
vesicle
cytosolic ribosome
membrane−bounded vesicle
−10*log10(p.adjusted)
E.
Non-Diff: DAD1 (ENST00000250498)
RA-Diff: DAD1 (ENST00000250498)
mRNA
RPF
Reads
100
50
0
25
50
AUG
STOP
−224
+0
+908
+1462

Figure S3

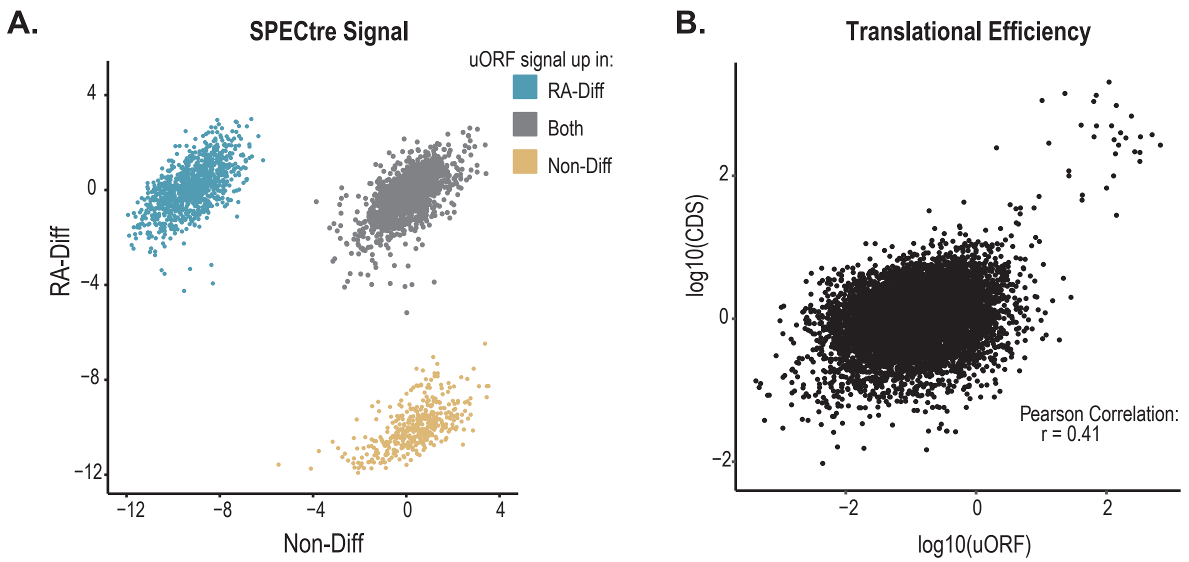
A.
SPECtre Signal
uORF signal up in:
RA-Diff
Both
Non-Diff
4
0
-4
-8
-12
RA-Diff
−12
−8
−4
0
4
Non-Diff
B.
Translational Efficiency
2
0
-2
log10(CDS)
−2
0
2
log10(uORF)
Pearson Correlation:
r = 0.41

Figure S4

Panther-GO Slim Biological Process	Raw p-value	FDR
protein-containing complex subunit organization	8.15E-09	1.46E-05
cellular component organization or biogenesis	2.38E-06	2.12E-03
cellular component organization	3.52E-06	2.09E-03
nucleosome assembly	1.84E-05	8.23E-03
regulation of actin filament polymerization	3.01E-05	1.07E-02
regulation of actin polymerization or depolymerization	4.81E-05	1.43E-02
actin filament polymerization	4.81E-05	1.23E-02
protein-DNA complex assembly	5.33E-05	1.19E-02
protein-DNA complex subunit organization	5.73E-05	1.14E-02
protein polymerization	5.75E-05	1.03E-02
ribonucleoprotein complex assembly	8.70E-05	1.41E-02
regulation of actin filament organization	9.48E-05	1.41E-02
ribonucleoprotein complex subunit organization	9.62E-05	1.32E-02
cellular protein complex disassembly	1.20E-04	1.53E-02
regulation of supramolecular fiber organization	1.61E-04	1.92E-02
protein-containing complex disassembly	1.87E-04	2.09E-02
negative regulation of actin filament polymerization	2.64E-04	2.77E-02

Figure S5

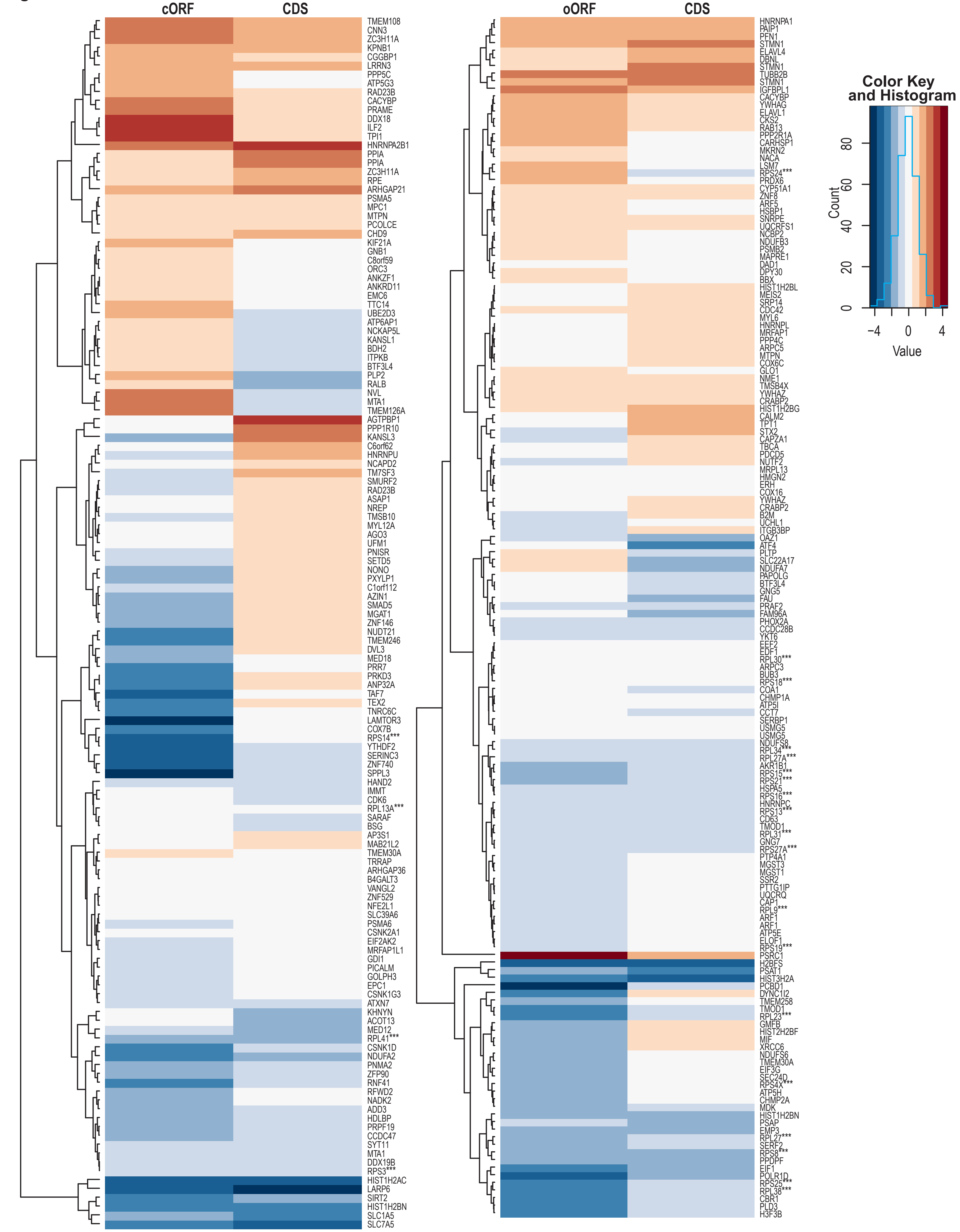

cORF
CDS
TMEM108
CNN3
ZC3H11A
KPNB1
CGGBP1
LRRN3
PPP5C
ATP5G3
RAD23B
CACYBP
PRAME
DDX18
ILF2
TPI1
HNRNPA2B1
PPIA
PPIA
ZC3H11A
RPE
ARHGAP21
PSMA5
MPC1
MTPN
PCOLCE
CHD9
KIF21A
GNB1
C8orf59
ORC3
ANKZF1
ANKRD11
EMC6
TTC14
UBE2D3
ATP6AP1
NCKAP5L
KANSL1
BDH2
ITPKB
BTF3L4
PLP2
RALB
NVL
MTA1
TMEM126A
AGTPBP1
PPP1R10
KANSL3
C6orf62
HNRNPU
NCAPD2
TM7SF3
SMURF2
RAD23B
ASAP1
NREP
TMSB10
MYL12A
AGO3
UFM1
PNISR
SETD5
NONO
PXYLP1
C1orf112
AZIN1
SMAD5
MGAT1
ZNF146
NUDT21
TMEM246
DVL3
MED18
PRR7
PRKD3
ANP32A
TAF7
TEX2
TNRC6C
LAMTOR3
COX7B
RPS14***
YTHDF2
SERINC3
ZNF740
SPPL3
HAND2
IMMT
CDK6
RPL13A***
SARAF
BSG
AP3S1
MAB21L2
TMEM30A
TRRAP
ARHGAP36
B4GALT3
VANGL2
ZNF529
NFE2L1
SLC39A6
PSMA6
CSNK2A1
EIF2AK2
MRFAP1L1
GDI1
PICALM
GOLPH3
EPC1
CSNK1G3
ATXN7
KHNYN
ACOT13
MED12
RPL41***
CSNK1D
NDUFA2
PNMA2
ZFP90
RNF41
RFWD2
NADK2
ADD3
HDLBP
PRPF19
CCDC47
SYT11
MTA1
DDX19B
RPS3***
HIST1H2AC
LARP6
SIRT2
HIST1H2BN
SLC1A5
SLC7A5
oORF
CDS
HNRNPA1
PAIP1
PFN1
STMN1
ELAVL4
DBNL
STMN1
TUBB2B
STMN1
IGFBPL1
CACYBP
YWHAG
ELAVL1
CKS2
RAB13
PPP2R1A
CARHSP1
MKRN2
NACA
LSM7
RPS24***
PRDX6
CYP51A1
ZNF8
ARF5
HSBP1
SNRPE
UQCRFS1
NCBP2
NDUFB3
PSMB2
MAPRE1
DAD1
DPY30
BBX
HIST1H2BL
MEIS2
SRP14
CDC42
MYL6
HNRNPL
MRFAP1
PPP4C
ARPC5
MTPN
COX6C
GLO1
NME1
TMSB4X
YWHAZ
CRABP2
HIST1H2BG
CALM2
TPT1
STX2
CAPZA1
TBCA
PDCD5
NUTF2
MRPL13
HMGN2
ERH
COX16
YWHAZ
CRABP2
B2M
UCHL1
ITGB3BP
OAZ1
ATF4
PLTP
SLC22A17
NDUFA7
PAPOLG
BTF3L4
GNG5
FAU
PRAF2
FAM96A
PHOX2A
CCDC28B
YKT6
EEF2
EDF1
RPL30***
ARPC3
BUB3
RPS18***
COA1
CHMP1A
ATP5I
CCT7
SERBP1
USMG5
USMG5
NDUFS8
RPL34***
RPL27A***
AKR1B1
RPS15***
RPS21***
HSPA5
RPS16***
HNRNPC
RPS13***
CD63
TMOD1
RPL31***
GNG7
RPS27A***
PTP4A1
MGST3
MGST1
SSR2
PTTG1IP
UQCRQ
CAP1
RPL9***
ARF1
ARF1
ATP5E
ELOF1
RPS19***
PSRC1
H2BFS
PSAT1
HIST3H2A
PCBD1
DYNC1I2
TMEM258
TMOD1
RPL23***
GMFB
HIST2H2BF
MIF
XRCC6
NDUFS6
TMEM30A
EIF3G
SEC24D
RPS4X***
ATP5H
CHMP2A
MDK
HIST1H2BN
PSAP
EMP3
RPL27***
SERF2
RPS8***
PPDPF
EIF1
POLR1D
RPS25***
RPL38***
CBR1
PLD3
H3F3B
Color Key
and Histogram
Count
0
20
40
60
80
−4
0
4
Value

Figure S6

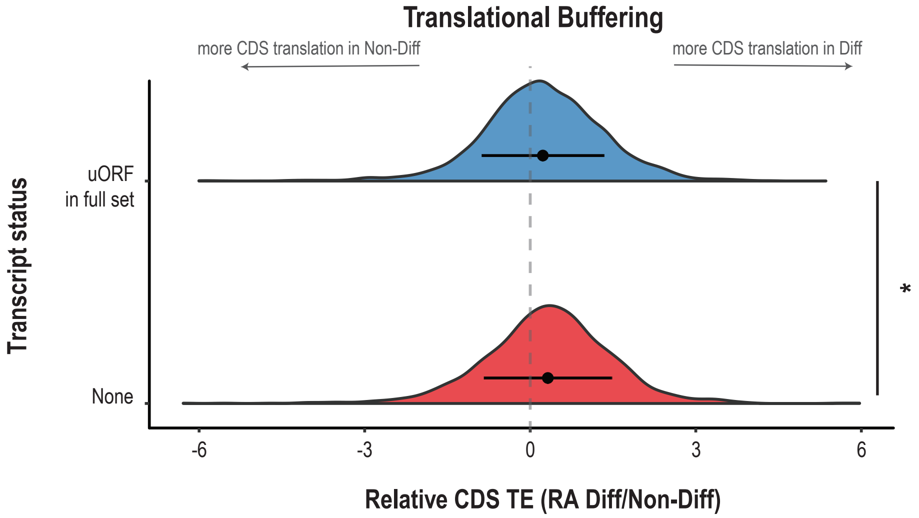

Translational Buffering
more CDS translation in Non-Diff
more CDS translation in Diff
Transcript status
uORF
in full set
None
*
-6
-3
0
3
6
Relative CDS TE (RA Diff/Non-Diff)
